# Scoring predictive models using a reduced representation of proteins: model and energy definition

**DOI:** 10.1186/1472-6807-7-15

**Published:** 2007-03-23

**Authors:** Federico Fogolari, Lidia Pieri, Agostino Dovier, Luca Bortolussi, Gilberto Giugliarelli, Alessandra Corazza, Gennaro Esposito, Paolo Viglino

**Affiliations:** 1 Dipartimento di Scienze e Tecnologie Biomediche, Università di Udine, P.le Kolbe 4, 33100 Udine, Italy; 2 INAF – Astronomical Observatory of Padova Vicolo dell'Osservatorio 5, I-35122 Padova, Italy; 3 Dipartimento di Matematica e Informatica, Università di Udine, Via delle Scienze 206, 33100 Udine, Italy; 4 Dipartimento di Fisica, Università di Udine, Via delle Scienze 206, 33100 Udine, Italy

## Abstract

**Background:**

Reduced representations of proteins have been playing a keyrole in the study of protein folding. Many such models are available, with different representation detail. Although the usefulness of many such models for structural bioinformatics applications has been demonstrated in recent years, there are few intermediate resolution models endowed with an energy model capable, for instance, of detecting native or native-like structures among decoy sets. The aim of the present work is to provide a discrete empirical potential for a reduced protein model termed here PC2CA, because it employs a PseudoCovalent structure with only 2 Centers of interactions per Amino acid, suitable for protein model quality assessment.

**Results:**

All protein structures in the set top500H have been converted in reduced form. The distribution of pseudobonds, pseudoangle, pseudodihedrals and distances between centers of interactions have been converted into potentials of mean force. A suitable reference distribution has been defined for non-bonded interactions which takes into account excluded volume effects and protein finite size. The correlation between adjacent main chain pseudodihedrals has been converted in an additional energetic term which is able to account for cooperative effects in secondary structure elements. Local energy surface exploration is performed in order to increase the robustness of the energy function.

**Conclusion:**

The model and the energy definition proposed have been tested on all the multiple decoys' sets in the Decoys'R'us database. The energetic model is able to recognize, for almost all sets, native-like structures (RMSD less than 2.0 Å). These results and those obtained in the blind CASP7 quality assessment experiment suggest that the model compares well with scoring potentials with finer granularity and could be useful for fast exploration of conformational space. Parameters are available at the url: .

## Background

Knowledge-based potential energy functions are extracted from protein structures. Most often a statistical analysis of database protein structures is performed. The potential involving a variable (e.g. a distance or an angle) is estimated from the distribution of that variable in the database, compared with that in a reference state or a null model [1-11]. Such potentials are often referred to as statistical effective energy functions (SEEFs).

Another class of knowledge-based potentials is based on optimization, that is the set of parameters for the potential functions are optimized, for instance, by maximizing the energy gap between the known native conformation and a set of alternative (or decoy) conformations [12-22]. This approach is strongly dependent on the methods used for building up decoys, and do not rely on an exact estimation of the energy gap existing between native and decoy structures.

The successful application of SEEFs to protein structure prediction tasks has been repeatedly demonstrated (see e.g. refs. [23,24]).

The statistical approach to the derivation of energy functions will be followed here. The structural representation of a protein ***s ***can be reduced to the coordinates of *C*_*α*_, *C*_*β*_, or side-chain centers which can be used to represent the location of a residue [25]. Once its amino acid sequence ***a ***is given, a function *f *mapping from the (***s***, ***a***) space to the *d*-dimensional space of descriptors is needed in order to allow a proper reduced protein description. A descriptor can be, e. g. the contact map between non-bonded residues, the solvent accessible surface area, a backbone or sidechain dihedral angle, the packing density and/or any other feature of protein structure. In practice the values that a variable (e.g. a residue-residue distance or an angle) can assume are discretized. The descriptors associated with that variable describe the possible discretized values of that variable and assume value 1 if the current value is within the bin associated to the descriptor and 0 otherwise. The potential function becomes therefore a map of the *d*-dimensional descriptors ***c ***to a real energy value. The energy is commonly computed as a linearly weighted sum of descriptors (for the notation we refer to ref. [26]):

H(f(s,a))=H(c)=w⋅c=∑iwici,
 MathType@MTEF@5@5@+=feaafiart1ev1aaatCvAUfKttLearuWrP9MDH5MBPbIqV92AaeXatLxBI9gBaebbnrfifHhDYfgasaacH8akY=wiFfYdH8Gipec8Eeeu0xXdbba9frFj0=OqFfea0dXdd9vqai=hGuQ8kuc9pgc9s8qqaq=dirpe0xb9q8qiLsFr0=vr0=vr0dc8meaabaqaciaacaGaaeqabaqabeGadaaakeaacqWGibascqGGOaakcqWGMbGzcqGGOaakieWacqWFZbWCcqGGSaalcqWFHbqycqGGPaqkcqGGPaqkcqGH9aqpcqWGibascqGGOaakcqWFJbWycqGGPaqkcqGH9aqpcqWF3bWDcqGHflY1cqWFJbWycqGH9aqpdaaeqbqaaiabdEha3naaBaaaleaacqWGPbqAaeqaaOGaem4yam2aaSbaaSqaaiabdMgaPbqabaGccqGGSaalaSqaaiabdMgaPbqab0GaeyyeIuoaaaa@4C92@

where "·" denotes inner product of vectors and *c*_*i *_is the number of occurrence of the *i*-th type of descriptor. For statistical knowledge-based potential functions, the weight vector ***w ***for linear potential is derived by characterization of the frequency distributions of structural descriptors in a database of experimentally determined protein structures.

### Statistical Effective Energy Functions

The Boltzmann's principle is usually invoked in order to obtain empirical free energies out of the observed statistical frequencies of various protein structural features, assumed to correspond to low energy states [1-3]. These energy functions can include pairwise contact terms [1] but also solvent terms [2], short-range and long-range pairwise interactions [3-5,27], dihedral angles [28,29], solvent accessibility, hydrogen-bonding [28] up to higher-order interactions [30,31] and three-body nonadditive interactions [32-34].

According to the Boltzmann principle, the distribution of protein molecules among the microscopic states at the equilibrium connects the potential function *H*(*γ*) for a microstate *γ *to its probability of occupancy *π*(*γ*). This probability *π*(*γ*) can be written as:

*π*(*γ*) = exp[-*H*(*γ*)/*kT*]/*Z*,

where *k *and *T *are the Boltzmann constant and the absolute temperature measured in Kelvin, respectively, and *Z *is the partition function. Following from Eq. (2) the knowledge-based potential function *H*(*γ*) corresponding to the Boltzmann distribution *π*(*γ*) is:

*H*(*γ*) = -*kT *ln *π*(*γ*) - *kT *ln *Z*.

In order to obtain a knowledge-based potential function, the background energetic interactions *H'*(*γ*) in the reference state must be defined. The effective potential energy is then:

ΔH(γ)=H(γ)−H′(γ)=−kTln⁡[π(γ)π′(γ)]−kTln⁡[ZZ′],
 MathType@MTEF@5@5@+=feaafiart1ev1aaatCvAUfKttLearuWrP9MDH5MBPbIqV92AaeXatLxBI9gBaebbnrfifHhDYfgasaacH8akY=wiFfYdH8Gipec8Eeeu0xXdbba9frFj0=OqFfea0dXdd9vqai=hGuQ8kuc9pgc9s8qqaq=dirpe0xb9q8qiLsFr0=vr0=vr0dc8meaabaqaciaacaGaaeqabaqabeGadaaakeaacqqHuoarcqWGibascqGGOaakiiGacqWFZoWzcqGGPaqkcqGH9aqpcqWGibascqGGOaakcqWFZoWzcqGGPaqkcqGHsislcuWGibasgaqbaiabcIcaOiab=n7aNjabcMcaPiabg2da9iabgkHiTiabdUgaRjabdsfaujGbcYgaSjabc6gaUjabcUfaBnaalaaabaGae8hWdaNaeiikaGIae83SdCMaeiykaKcabaGaf8hWdaNbauaacqGGOaakcqWFZoWzcqGGPaqkaaGaeiyxa0LaeyOeI0Iaem4AaSMaemivaqLagiiBaWMaeiOBa4Maei4waS1aaSaaaeaacqWGAbGwaeaacuWGAbGwgaqbaaaacqGGDbqxcqGGSaalaaa@5D9A@

where *π'*(*γ*) is the probability of the descriptor *γ *in the reference state. *Z *and *Z' *are both constants. Following Sipple, it is usually assumed that *Z *≈ *Z'*, so that Eq. (4) becomes [3]:

ΔH(γ)=−kTln⁡[π(γ)π′(γ)]
 MathType@MTEF@5@5@+=feaafiart1ev1aaatCvAUfKttLearuWrP9MDH5MBPbIqV92AaeXatLxBI9gBaebbnrfifHhDYfgasaacH8akY=wiFfYdH8Gipec8Eeeu0xXdbba9frFj0=OqFfea0dXdd9vqai=hGuQ8kuc9pgc9s8qqaq=dirpe0xb9q8qiLsFr0=vr0=vr0dc8meaabaqaciaacaGaaeqabaqabeGadaaakeaacqqHuoarcqWGibascqGGOaakiiGacqWFZoWzcqGGPaqkcqGH9aqpcqGHsislcqWGRbWAcqWGubavcyGGSbaBcqGGUbGBcqGGBbWwdaWcaaqaaiab=b8aWjabcIcaOiab=n7aNjabcMcaPaqaaiqb=b8aWzaafaGaeiikaGIae83SdCMaeiykaKcaaiabc2faDbaa@4688@

Due to the high dimensionality of the space of descriptors a reasonable factorization of the probability ratio is sought. Different variables are typically treated independently of each other, resulting thus in an energy function that is the sum of many independent contributions. For each descriptor *c*_*i *_the contribution to the energy is given by *w*_*i *_= -*kT *ln[*π*(*c*_*i*_)/*π'*(*c*_*i*_)]. Whether they are formalized in a discretized or analytical way, contibutions to the energy functions derived in this way are technically potentials of mean force [35]. The effectiveness and the drawbacks of the approach have been repeatedly pointed out [32]. First, it is straightfoward that the choice of the reference state is critical for developing knowledge-based statistical potential function. The reference state problem has been clearly stated by Jernigan and Bahar [10] and discussed by Skolnick and coworkers [36,37] who derived a potential for a compact reference state with a bias for buried hydrophobic residues and compared it with previous contact potentials.

Second the choice of descriptor must catch most aspects of protein energetics, i.e. only native-like models should be assigned low energy by the potential.

Third, the assumption of independence of different potentials of mean force is clearly unrealistic. Notwithstanding all limitations, statistical effective energy functions are currently the most successful potential available for describing protein conformations (see e.g. refs. [23,24]).

Many Statistical Effective Energy Functions have been derived according to different level of representation of the protein, the features selected for defining the potential, the reference state and the actual way of derivation of the potential. Since excellent reviews on this subject are available in the literature we will not attempt an extensive coverage of all the works published so far, but rather we aim at discussing general aspects on this issue with respect to the work presented here [8,38-40].

Earlier works focused on the preference of contacts between specific residues in the database of available experimental structures [1,2,41-43]. These and other works pointed out that preferential interactions are one of the most relevant features that must be taken into account for describing protein energetics [38].

The demonstrated unlearnability of optimal potential on simplified models of proteins points out that most likely [17]:

i) the chosen protein representation requires a higher level of detail;

ii) the actual residue-residue contact definition is crucial for the accuracy of the potential [44]

iii) other features, like local conformational preferences, must be taken into account for discriminating native structure among decoys;

Another important result, which is worth mentioning here, obtained by Park and Levitt is that smoothing the contact energy function improves the performance of the potential in native structure discrimination among decoys [45].

The derivation of statistical potentials requires the definition of a reference state. The finite size of database proteins and the diversity of sidechains makes this task not straightforward. This problem has been repeatedly pointed out and recently novel approaches have been proposed [10,27].

Concerning other features, like local conformational preferences, it is well known that such preferences exist and actually they form the basis of the success of secondary structure prediction algorithms [46] and current single sequence algorithm are able to predict secondary structure with a 3-state accuracy of 70.3% [47].

Recently convincing evidence has been provided that, for high resolution structures, the distribution of backbone and sidechain dihedral angles may be used for assessing the quality of predictive models [48-50] and may be successfully used for supplementing contact potentials (see e. g. ref. [51]). Moreover it has been pointed out that dihedral angles are strongly correlated with the identity of adjacent residues [52].

The development of the potential presented here was motivated by lack of a reliable potential employing a coarse grained representation of protein structures with the following features:

- only two (or one for glycine) centers of interaction per residues;

- smooth interactions e.g. by binning a range interval;

- off-lattice representation with a continuum range for all conformational variables;

- inclusion of residue-dependent local conformational potential term favoring observed preferences;

- inclusion of (at least) nearest neighbor correlation which could reproduce local conformation correlation.

This term is fundamental for obtaining the proper average length of helices, similar to nearest-neighbor coupling in one-dimensional Ising models and it is not usually considered in available potentials.

Although most of the above listed elements are found in available empirical potentials, an empirical potential that includes all these features at the same time is missing in the literature. There are a number of empirical potentials available, which are similar in spirit. For instance a similar model, but including up to three centers of interactions per sidechain, has been used successfully by Betancourt [53]. The distance-based potential developed by Zhou and coworkers achieves good performance with an even coarser grained representation [54].

Other potentials include structural features of interaction centers like orientational parameters for interactions (see e.g. the UNRES potential [55-57] and the review by [40]). Other potentials, used for simplified model simulations in physics, include all backbone atoms as reviewed by [38]. The statistical potential of Dehouck et al. includes all backbone torsion angles (broadly classified into seven classes) and a center of interaction for each sidechain [31].

Usage of the correlation between local amino acid conformations has been shown to improve the native structure recognition capability of scoring functions by [48]. Recently correlation between different sequence and structure descriptors (associated with potentials of mean force) has been built in a general framework by Dehouck and coworkers. The best SEEF developed according to this framework, entailing 30 energy terms, compares well or better with most popular SEEFs [31].

The reduced representation proposed here consists of two centers of interaction per residue, one for the backbone, centered on C_*α *_atom and one for the sidechain (except for glycine which entails only one center of interaction for the backbone) centered on the center of mass of the sidechain atoms.

The potential function entails energy terms for the pseudo bonds between two consecutive C_*α*_'s and between a C_*α *_and the center of mass of the relative sidechain, angular energy terms for all three pseudo-bonded centers of interaction, torsional energy terms for all four pseudo-bonded centers of interaction, a pseudo-torsional energy terms to mantain proper chirality of sidechain orientation with respect to the main chain, an energy term dependent on the torsional angles of adjacent quartets of consecutive C_*α*_'s and energy terms for all pairwise non-bonded interactions. The pairwise interactions between centers of interaction is derived here from database analysis and it employs a reference state which takes into account the finite size of proteins. The model and energy definition are detailed in the Materials and methods section.

We termed this reduced representation of proteins **PC2CA**, an acronym for **P**seudo**C**ovalent structure with **2 C**enters of interaction per **A**mino acid.

The performance of this potential on all multiple decoy sets in the Decoys'R'us database [58] is tested and the results obtained in the Critical Assessment of Structural Predictions (CASP7) model Quality Assessment (QA) category are summarized.

## Results

### Analysis of the different energy terms

The average and standard deviation values of the different energy terms have been evaluated on the top500H database structures. For what concerns the covalent energy terms (terms *i *to *vi *in Eq. (6)), only few structures, notably with short sequences, had significant deviations in the energy terms connected with the positioning of the sidechain center of mass (terms *ii *to *iv *in equation 6).

Particular attention has been paid to the torsional term dependent on the local chain conformation and the term describing the correlation between adjacent local conformations. In order to make sure that these two terms could describe reasonably well the known preferences for secondary structure elements, a Monte Carlo simulation was run on the sequences of all the structures in the top500H datasets. The energy was just the sum of the torsional (term *vii *in equation 7) and correlation energy (term *viii *in equation 8) and the temperature factor *kT *was set to 1.0 in order to match the derivation of the potential.

The range 30 to 70 degrees was assigned to helical conformation, the range 170 to 240 was assigned to extended conformation and all other conformations were assigned to coil conformations. Every residue was assigned to the most populated conformational range (actually averaged on the preceding and the following bond, because all torsional angles refer to bonds involving two adjacent residues). The experimental secondary structure was obtained using the program DSSP [59] and converting the result into three states (extended, helix, coil): the 'E' state was left as the extended conformation; the 'H', 'G' and 'I' states were converted into helix, and all other states into coil. No post-processing of the results was performed.

Although this test is run on the same structures used for deriving the potential, making it invalid for any quantitative assessment, the three-state accuracy of this simple prediction procedure is 0.57 which is more or less what expected for a single sequence method using only nearest neighbor information.

It is interesting to assess the relative contribution of the correlation term to this accuracy. The same test has been repeated setting the correlation term to zero. The accuracy dropped to 0.51. This was a confirmation that the correlation term is indeed important for properly reproducing local conformational propensities.

### Test on the multiple decoy datasets

The decoy sets available in the Decoys'R'us database under the category 'multiple' are sets where many alternative conformations are given for a single native structure. The models are obtained with widely different methods and offer therefore a significant challenge for free energy estimators. The sets have different features which make different measures of performance appropriate. The ten sets which are currently available are shortly described hereafter.

The set 4state_reduced contains alternative models for 7 different proteins. For each protein native-like conformations are present in the set and therefore some correlation between rmsd and energy should witness the accuracy of the energy function [45].

The two sets fisa and fisa_casp3 have been assembled by the group of Baker using fragments via a simulated annealing protocol [60]. For the protein 130 the structure with pdb code 1ck2 has been used as the native structure.

The sets ig_structal, hg_structal and ig_structal_hires are sets containing few models for many immunoglobulins (ig) or globins (hg) built by homology modeling. Most of the models have very low RMSD from native.

The set lattice_ssfit has been generated selecting and refining with an all-atom energy function coarse lattice models [61]. The RMSD from native in the set is larger than 4 Å for all the eight proteins modeled.

The lmds set was built including information on secondary structure and models have been refined using a soft core all atom model [62]. The set includes models with RMSD from native lower than 5.0 Å for 10 proteins.

The semfold test has been produced apparently by a fragment insertion method [63]. This includes a very large number (average of 12900) of decoys for each of the 6 proteins. Some models have RMSDs from native in the range 3 to 5 Å.

The vhp_mcmd decoy set has been obtained by taking snapshots of long molecular dynamics simulations starting from the native structure and from four coarse grained models obtained by Monte Carlo simulations. All conformations have been energy minimized using the molecular mechanics/generalized Born model [51].

The results obtained on the decoy sets are summarized in tables 1 to 10. Since many of the target structures have homologues in the top500H database, those which do not have a significant similarity with the top500H set are indicated by boldface characters (we used as a criterion an E-value greater than 0.01 for the best alignment with BLAST). No significant difference is apparent based on the presence or absence of homologues in the top500H dataset.

**Figure 1:**
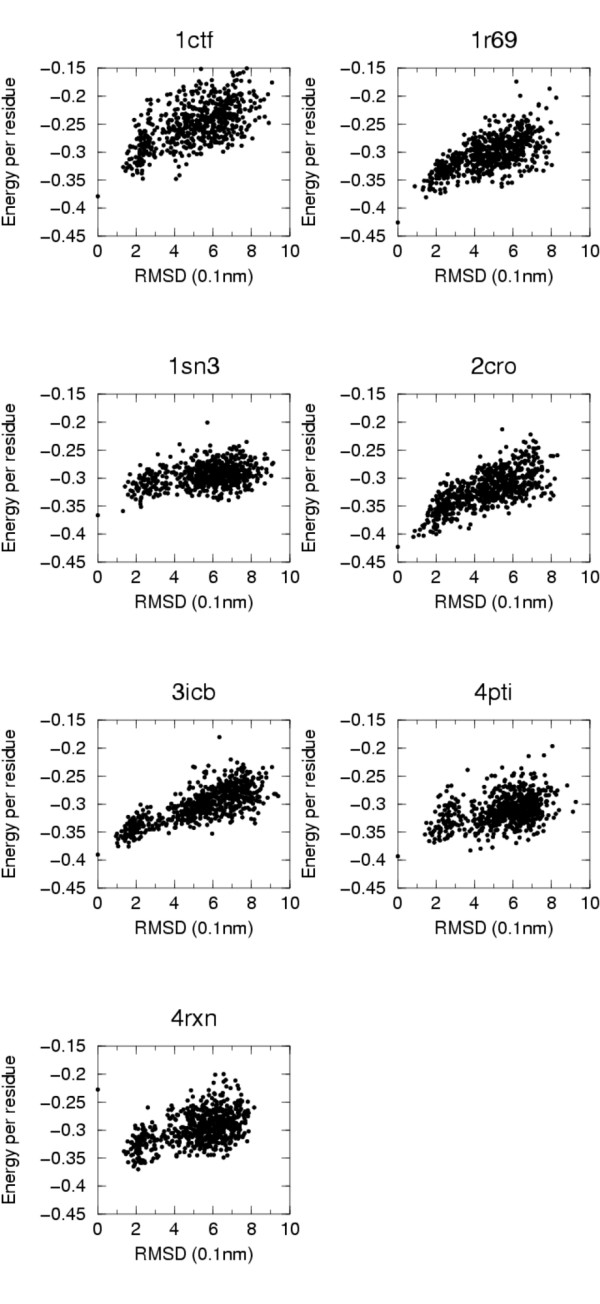
Energy versus RMSD plots for the 4state decoy sets.

An energy versus RMSD plot for the 4state decoy sets is reported in Figure 1.

There are a number of plausible reasons for failure in native structure recognition, even for the best quality scoring functions, as discussed by Shen and Sali [64]. In the present case, in the few cases where the native structure is not recognized the covalent energy of the pseudocovalent structure is large showing that most likely the experimental model is not optimally refined. For instance in the native structure of protein with PDB code 4rxn there the distance between the C_*α *_of Glu 16 and the C_*α *_of Asp 17 is 3.19 Å, much shorter than the average distance causing the highest energy in the set for the corresponding energy term. Similarly there are distorted geometries in the proteins with PDB code 1ctf, 1bba and 1dtk.

Another likely reason for failure of native structure recognition is the presence in the crystal structure of other chains. This is the case for the short fragment of protein A (chain C of PDB structure with code 1fc2) which is bound in the crystal to an immunoglobulin domain.

In general NMR structures are more difficult to be recognized. Refinement of NMR structures is strongly dependent on the forcefield and protocol used, and this may result in minor structural features which are not typical of protein structures and give rise, in turn, to large energies in the statistical effective energy function.

The most challenging decoy set appears to be the semfold set which includes six targets and more than 10000 decoys for each target. For five of the decoys native or low RMSD decoys could be recognized, but the Z-score is rather low. For the structure with PDB code 1khm the lowest energy structure has a high RMSD from native, although there are decoys with RMSD from native as low as 3.0 or 4.0 Å. It is remarkable that the term for CA CM interactions attains the third lowest energy for the native structure but the overall energy is only the tenth lowest energy beyond structures with RMSD from native larger than 10 Å. The number of native-like structures (say with RMSD from native less than 4.0 Å) in the set is however limited, so it is difficult to assess whether the failure can be ascribed to the quality of the native structure, of the decoys or of the energy function itself. For this reason we considered other structures deposited in the PDB with the same sequence. For the structure with PDB code 1zzj the energy (computed on the same fragment modeled in the decoy set) is lower than the energy of all decoys. This result witnesses the quality of the energy function although the low energy assigned to very different conformations poses an issue on the robustness of the methodology. It is worth mentioning that the protein is associated with single stranded RNA in the crystallographic structure, and this feature is not modelled by the statistical effective potential.

The energy function performs also well on decoys which are mostly native-like as in the decoy sets hg_structal, ig_structal, ig_structal_hires where the native conformation is recognized 13 times on 110 cases.

Moreover very low RMSD decoys are mostly selected as the lowest energy conformations. For this sets it is interesting that the correlation coefficient between energy and RMSD is on average high (0.44) and for the hg_structal set it is on average equal to 0.71.

The correlation between energy and RMSD is typically found only at low RMSDs, in other words the energy for grossly misfolded structures is not correlated with the RMSD from the native structure, but should be correlated with the RMSD from the local minimum energy conformation. For sets where the whole range of RMSDs is represented the correlation coefficient should be positive and significantly different from 0.0 and similarly the fraction enrichment should be significantly larger than 10 percent. Indeed this is the case for all 4state_reduced decoy sets and for the vhp_mcmd sets, and on average for the hg_structal, ig_structal, ig_structal_hires.

The overall ability of recognizing the native structure among decoys including native-like structures is outstanding for a model entailing only two centers of interaction per amino acid, and compares well or is superior to more complex models as judged by the results obtained with many model quality assessment programs and reported by Tosatto (see Table 3 in [50]). We report in table 11 a summary of the data reported in the cited study by Tosatto including the best performing potentials together with our results, for the sake of comparison. The best Model Quality Assessment Programs (MQAPs) considered here are ProQ [65], Prosa II [66], Verify3d [67,68], AKBP [5], DFIRE [27], RAPDF [4] and FRST [50].

### Test on conformations generated by Rosetta

A test was performed by generating 100 conformations for the small thermostable domain of the chicken villin headpiece using the software Rosetta which is one of the best tools for ab-initio protein structure prediction. 100 structures have been generated and refined using the same software. The average RMSD of the conformations with respect to native is 3.6 Å with a standard deviation of 1.1 Å. The best model selected by the energy model proposed here has 3.0 Å RMSD from native. Perhaps more significant is the correlation between the rank according to the energy and the rank according to the RMSD which is 0.48.

### PC2CA in CASP7

At the time this paper is being written the CASP7 experiment has just closed (for a description of the CASP experiment see ref. [69]). For 99 out of the 100 targets experimental structures have been released. The quality assessement category has been recently introduced in the CASP experiment in order to evaluate by scoring functions the quality of the predictive models obtained by servers. The discussion reported hereafter is connected with the methods adopted for assessment in this community-wide experiment (see ref. [70] and forthcoming articles in Proteins: Structure, Function, Bioinformatics).

Unfortunately models submitted by servers and evaluated by quality assessement programs differ in the number of residues modeled and in the level of detail, ranging from only C_*α*_'s to all atoms for each amino acid. For this reason a choice must be adopted for ranking the models which takes into account these aspects.

Evaluation of predictions may be conducted using different legitimate criteria. We discuss in the following the ability of PC2CA to select native-like structures among decoys.

In order to assess the performance of PC2CA we evaluate the quality of best ranking models using the widely accepted Global Distance Test Total Score (GDT_TS) criterion [71]. In particular the "loss in GDT_TS" of the best ranked model (i.e. lowest energy model) compared to the best available model (according to GDT_TS) (see A. Tramontano's presentation at CASP7 available at [70]) gives a good idea of the performance of an energy or scoring model.

We wish to remark that no selection has been applied nor on targets nor on models: all models were scored for all targets by our group. No consensus method nor alignment or template modeling has been used for scoring models. The average loss in GDT_TS may be greatly reduced by selecting homology modeling targets. Considering the consensus among predictors improves results in many categories of predictions, as demonstrated in recent CASP experiments.

Another important issue is that residues with incomplete backbone or sidechain have been simply ignored in our quality assessment predictions and therefore a large number of models received very low score. In the deposited quality assessments we ranked models according to the energy per residue with a cutoff on the percentage of modeled residues for half of the targets and according to global energy for the remaining half. The energies for models with different level of completeness are not directly comparable and the chosen criterion had only the purpose to single out best and most complete models.

Here we will discuss predictions based on the global energy, but the results described here are however largely overlapping with those deposited.

The presence of heterogeneous (regards to completeness in length and heavy atoms) predictions made the correlation between ranking and GDT_TS insignificant and, in general, it impaired safe comparison of models.

The global energy appeared a reasonable criterion for scoring best models but it was not designed in order to maximize correlation of score rank with GDT_TS rank.

When the GDT_TS of the best models obtained from servers is compared with the GDT_TS of the best scoring model according to PC2CA the results are outstanding when one considers that the energy model employs only two centers of interaction per residue. The average loss in GDT_TS is 10.3 for all targets (10.0 and 11.9 for template modeling and template free modeling targets according to the assessors' classification, respectively). When the best scoring model is compared to the first model submitted by most successful servers like Zhang server and ROBETTA server the difference in GDT_TS is as low as 5.4 and 1.5, respectively, and in general the average loss in GDT_TS compared to the best MQAP predictor is 4.7. Comparison with other MQAP predictors in CASP7 is not straightforward because only 7 groups (including ours) deposited predictions for all targets. When we compare the average GDT_TS of the best PC2CA scoring models with that of other predictors (with the average performed on the same predictions deposited by each group) PC2CA ranks 15th out of 26 for template free modeling and 18th out of 26 for all models. The average loss in GDT_TS computed is 10.1, smaller than the average of 10.7 of all quality assessment methods.

**Figure 2:**
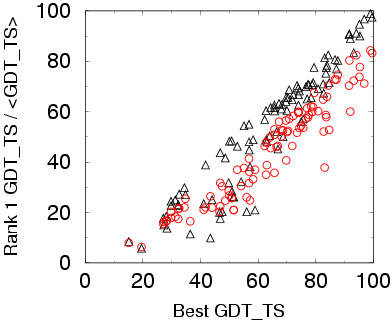
GDT_TS of the best PC2CA scoring models (black triangles)and average GDT_TS of predictive models (red circles) versus GDT_TS of the best predictive model for each target in CASP7 MQAP.

The best PC2CA scoring models have consistently higher GDT_TS than the average GDT_TS computed on all models for each target (Figure 2).

## Discussion

The reduced representation of proteins presented in this work has two features which makes it attractive for application in biophysical areas: it is simple and it is capable of discerning native-like models among non native-like models produced using a wide variety of methods. A blind test performed in the CASP7 quality assessment category confirms this conclusion and, in spite of its granularity, our scoring potential ranks in the average of methods using finer granularity and applying consensus procedures.

The good scoring properties of the model however do not guarantee that the same model can be used for folding small proteins e. g. by Monte Carlo simulated annealing. Indeed, the potential could have lower minima for conformations that are not explored by the algorithms used for generating decoys or predictive models in CASP7. The range of values for terms involving bonds, angles and pseudodihedrals in the systems tested is limited. It is likely that it would be possible to slightly increase the energy of these terms, reaching non-physical conformations, and simultaneously decrease other, e. g. non-bonded energy terms.

An obvious correction to the potential for model generation applications is the replacement of flat high energy regions at large distances with increasing potential, in order to prevent bonds to break. A less obvious issue is that the weights of the different terms, in particular those which have less variability in the decoy test sets, could have to be changed when using the potential to generate models. It is also likely that the correlation found between torsional and bending energy terms in native structures will need proper treatment. Such correlations are somehow taken into account by the attractive potential between CA's.

Another important point is that the pseudo-minimization used in this work is a very rough approximation of a real energy minimization procedure.

The ultimate test for a scoring potential function is however recognition of native-like structures among decoys generated with the task of minimizing the same potential function.

All these issues above are being considered for using the potential in model generation applications under development in our laboratories.

## Conclusion

The results obtained on decoy sets and those obtained in the blind CASP7 quality assessment experiment suggest that the energetic model proposed here is suited for scoring predictive protein models. In spite of its simplicity the potential compares well with scoring potentials with much finer granularity. Tests are underway in order to assess its application for fast exploration of conformational space.

## Methods

### Selection of a protein dataset

The dataset of proteins used for the extraction of the statistical potential is Top500H [49]. This is a non-redundant set of 500 hand-cured proteins resolved by X-ray cristallography to 1.8 Å or better resolution. There are few Van der Waals clashes and few significant deviations from ideal bond lenghts and angles and a maximum of 60% sequence identity is allowed for each structure pair.

### Derivation of a statistical potential

All protein structures in the Top500H dataset have been read and converted in reduced form including only C_*α *_atoms (CA), representing the backbone atoms, and the center of mass of each sidechain representing the sidechain itself (CM). Adjacency between amino acids was checked by the requirement that, after sorting residue numbers and residue insertions in the PDB file the distance between consecutive C_*α*_'s was in the range 2.7 to 4.3 Å. Based on this reduced model a statistical effective energy function has been derived from the analysis of the Top500H dataset. Incomplete backbone groups or sidechains have been discarded from analysis.

**Figure 3:**
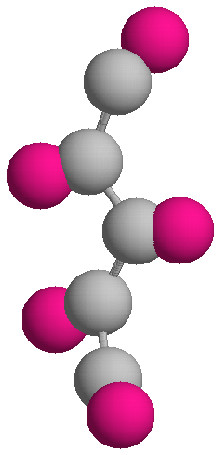
Pseudocovalent structure for a five-residue fragment of a protein (PDB id 1CTF, fragment 54–58).

For this reduced model a pseudocovalent structure may be defined where each CA is bonded to its sidechain center of mass (CM) and to the adjacent CAs [25] (Figure 3). Consequently, following the standard terminology for molecular mechanics forcefields, the energy model entails bonded and non-bonded terms. CM is absent for glycine residues.

The energy linked to the quality of the pseudocovalent structure *E*_*cov*_, rather than to the quality of the conformation itself, is described by the sum of six terms (each implying summation on index *i*):

Ecov=i)Eb(d(CA(i),CA(i+1)))+ii)Eb(d(CA(i),CM(i)))+iii)12Ea(θ(CA(i),CA(i+1),CM(i+1)))+iv)12Ea(θ(CM(i),CA(i),CA(i+1)))+v)Ea(θ(CA(i),CA(i+1),CA(i+2))))+vi)Ep(φ(CA(i),CA(i+1),CA(i+2),CM(i+1))))
 MathType@MTEF@5@5@+=feaafiart1ev1aaatCvAUfKttLearuWrP9MDH5MBPbIqV92AaeXatLxBI9gBaebbnrfifHhDYfgasaacH8akY=wiFfYdH8Gipec8Eeeu0xXdbba9frFj0=OqFfea0dXdd9vqai=hGuQ8kuc9pgc9s8qqaq=dirpe0xb9q8qiLsFr0=vr0=vr0dc8meaabaqaciaacaGaaeqabaqabeGadaaakeaafaqaaaGbeaaaaaqaaiabdweafnaaBaaaleaaieGacqWFJbWycqWFVbWBcqWF2bGDaeqaaaGcbaGaeyypa0dabaGaemyAaKMaeiykaKcabaGaemyrau0aaSbaaSqaaiabdkgaIbqabaGccqGGOaakcqWGKbazcqGGOaakcqWGdbWqcqWGbbqqcqGGOaakcqWGPbqAcqGGPaqkcqGGSaalcqWGdbWqcqWGbbqqcqGGOaakcqWGPbqAcqGHRaWkcqaIXaqmcqGGPaqkcqGGPaqkcqGGPaqkcqGHRaWkaeaaaeaaaeaacqWGPbqAcqWGPbqAcqGGPaqkaeaacqWGfbqrdaWgaaWcbaGaemOyaigabeaakiabcIcaOiabdsgaKjabcIcaOiabdoeadjabdgeabjabcIcaOiabdMgaPjabcMcaPiabcYcaSiabdoeadjabd2eanjabcIcaOiabdMgaPjabcMcaPiabcMcaPiabcMcaPiabgUcaRaqaaaqaaaqaaiabdMgaPjabdMgaPjabdMgaPjabcMcaPaqaamaalaaabaGaeGymaedabaGaeGOmaidaaiabdweafnaaBaaaleaacqWGHbqyaeqaaOGaeiikaGccciGae4hUdeNaeiikaGIaem4qamKaemyqaeKaeiikaGIaemyAaKMaeiykaKIaeiilaWIaem4qamKaemyqaeKaeiikaGIaemyAaKMaey4kaSIaeGymaeJaeiykaKIaeiilaWIaem4qamKaemyta0KaeiikaGIaemyAaKMaey4kaSIaeGymaeJaeiykaKIaeiykaKIaeiykaKIaey4kaScabaaabaaabaGaemyAaKMaemODayNaeiykaKcabaWaaSaaaeaacqaIXaqmaeaacqaIYaGmaaGaemyrau0aaSbaaSqaaiabdggaHbqabaGccqGGOaakcqGF4oqCcqGGOaakcqWGdbWqcqWGnbqtcqGGOaakcqWGPbqAcqGGPaqkcqGGSaalcqWGdbWqcqWGbbqqcqGGOaakcqWGPbqAcqGGPaqkcqGGSaalcqWGdbWqcqWGbbqqcqGGOaakcqWGPbqAcqGHRaWkcqaIXaqmcqGGPaqkcqGGPaqkcqGGPaqkcqGHRaWkaeaaaeaaaeaacqWG2bGDcqGGPaqkaeaacqWGfbqrdaWgaaWcbaGaemyyaegabeaakiabcIcaOiab+H7aXjabcIcaOiabdoeadjabdgeabjabcIcaOiabdMgaPjabcMcaPiabcYcaSiabdoeadjabdgeabjabcIcaOiabdMgaPjabgUcaRiabigdaXiabcMcaPiabcYcaSiabdoeadjabdgeabjabcIcaOiabdMgaPjabgUcaRiabikdaYiabcMcaPiabcMcaPiabcMcaPiabcMcaPiabgUcaRaqaaaqaaaqaaiabdAha2jabdMgaPjabcMcaPaqaaiabdweafnaaBaaaleaacqWGWbaCaeqaaOGaeiikaGIae4NXdyMaeiikaGIaem4qamKaemyqaeKaeiikaGIaemyAaKMaeiykaKIaeiilaWIaem4qamKaemyqaeKaeiikaGIaemyAaKMaey4kaSIaeGymaeJaeiykaKIaeiilaWIaem4qamKaemyqaeKaeiikaGIaemyAaKMaey4kaSIaeGOmaiJaeiykaKIaeiilaWIaem4qamKaemyta0KaeiikaGIaemyAaKMaey4kaSIaeGymaeJaeiykaKIaeiykaKIaeiykaKIaeiykaKcaaaaa@F102@

where the subscripts *b*, *a *and *p *refer to bond, angle and pseudodihedral energies, respectively.

The domain of each bond, angle and pseudodihedral variable considered has been divided into bins and the energy corresponding to the *k*^*th *^bin has been obtained as *E*(*k*) = -*log*(NkNtot
 MathType@MTEF@5@5@+=feaafiart1ev1aaatCvAUfKttLearuWrP9MDH5MBPbIqV92AaeXatLxBI9gBaebbnrfifHhDYfgasaacH8akY=wiFfYdH8Gipec8Eeeu0xXdbba9frFj0=OqFfea0dXdd9vqai=hGuQ8kuc9pgc9s8qqaq=dirpe0xb9q8qiLsFr0=vr0=vr0dc8meaabaqaciaacaGaaeqabaqabeGadaaakeaadaWcaaqaaiabd6eaonaaBaaaleaacqWGRbWAaeqaaaGcbaGaemOta40aaSbaaSqaaiabdsha0jabd+gaVjabdsha0bqabaaaaaaa@3510@), where *N*_*k *_is the number of counts in the *k*^*th *^bin and *N*_*tot *_is the number of total counts. Intervals with *N*_*k *_= 0 were assigned arbitrarily a value *E*(*k*) = -2*log*(1Ntot
 MathType@MTEF@5@5@+=feaafiart1ev1aaatCvAUfKttLearuWrP9MDH5MBPbIqV92AaeXatLxBI9gBaebbnrfifHhDYfgasaacH8akY=wiFfYdH8Gipec8Eeeu0xXdbba9frFj0=OqFfea0dXdd9vqai=hGuQ8kuc9pgc9s8qqaq=dirpe0xb9q8qiLsFr0=vr0=vr0dc8meaabaqaciaacaGaaeqabaqabeGadaaakeaadaWcaaqaaiabigdaXaqaaiabd6eaonaaBaaaleaacqWG0baDcqWGVbWBcqWG0baDaeqaaaaaaaa@3346@), which is equivalent to the usage of pseudocounts (1Ntot
 MathType@MTEF@5@5@+=feaafiart1ev1aaatCvAUfKttLearuWrP9MDH5MBPbIqV92AaeXatLxBI9gBaebbnrfifHhDYfgasaacH8akY=wiFfYdH8Gipec8Eeeu0xXdbba9frFj0=OqFfea0dXdd9vqai=hGuQ8kuc9pgc9s8qqaq=dirpe0xb9q8qiLsFr0=vr0=vr0dc8meaabaqaciaacaGaaeqabaqabeGadaaakeaadaWcaaqaaiabigdaXaqaaiabd6eaonaaBaaaleaacqWG0baDcqWGVbWBcqWG0baDaeqaaaaaaaa@3346@). The width of the bins and particular cases will be discussed in the following.

The first term (*i*) is the energy associated with the bond between two consecutive CAs. The distribution of the CA-CA distance depends essentially on the presence or absence of a proline in the second position and therefore only two distributions (with proline or any other residue at the second position, respectively) have been considered. The bins are 0.025 Å wide. The second term (*ii*) is the energy associated with the bonds between CA and the sidechain center of mass CM. This energy term is specific for all amino acid types (other than glycine). The bins considered are 0.1 Å wide. The terms (*iii*) and (*iv*) represent the energy associated with the angles between the CA-CM vector and the preceding and following CA-CA vectors, respectively. These terms are specific for the amino acid type of the residue involving the CA-CM vector. The bins are 5 degrees wide. The 12
 MathType@MTEF@5@5@+=feaafiart1ev1aaatCvAUfKttLearuWrP9MDH5MBPbIqV92AaeXatLxBI9gBaebbnrfifHhDYfgasaacH8akY=wiFfYdH8Gipec8Eeeu0xXdbba9frFj0=OqFfea0dXdd9vqai=hGuQ8kuc9pgc9s8qqaq=dirpe0xb9q8qiLsFr0=vr0=vr0dc8meaabaqaciaacaGaaeqabaqabeGadaaakeaadaWcaaqaaiabigdaXaqaaiabikdaYaaaaaa@2E9E@ factor is used because the terms (*iii*) to (*vi*) are not independent and clearly a single angular term involving CM would suffice to restrain the position of CM with respect to the trace of the protein.

The 12
 MathType@MTEF@5@5@+=feaafiart1ev1aaatCvAUfKttLearuWrP9MDH5MBPbIqV92AaeXatLxBI9gBaebbnrfifHhDYfgasaacH8akY=wiFfYdH8Gipec8Eeeu0xXdbba9frFj0=OqFfea0dXdd9vqai=hGuQ8kuc9pgc9s8qqaq=dirpe0xb9q8qiLsFr0=vr0=vr0dc8meaabaqaciaacaGaaeqabaqabeGadaaakeaadaWcaaqaaiabigdaXaqaaiabikdaYaaaaaa@2E9E@ factor in terms (*iii*) and (*iv*) does not apply for terms involving the ends of the chain.

The term (*v*) represents the energy associated with the angle among three consecutive CAs. The last term (*vi*) is an energy term associated with the pseudodihedral defined by three consecutive CAs and the sidechain center of mass of the second residue. This term is needed in order to mantain the proper orientation of the sidechain center of mass with respect to the main chain plane.

Both terms (*v*) and (*vi*) are specific for the central amino acid type. The bins considered are 5 degrees wide. 

The sequence local conformational propensity is taken into account with an energy which depends on the dihedral angle defined by four consecutive CAs

*E*_*dih *_= *vii*) *E*_*t*_(*φ*(*CA*(*i*), *CA*(*i *+ 1), *CA*(*i *+ 2), *CA*(*i *+ 3)))

where the subscript *t *refers to torsional energies. The bins considered here are 10 degree wide.

This term is specific for the two central amino acid types, i.e. there are 400 different potentials of mean force defined.

In order to take into account properly the occurrence of secondary structure elements and other frequent local conformation motifs, like turns, an additional term describes the correlation between adjacent local conformations:

*E*_*corr *_= *viii*) *E*_*c*_(*φ*(*CA*(*i*), *CA*(*i *+ 1), *CA*(*i *+ 2), *CA*(*i *+ 3)), *φ*(*CA*(*i *+ 1), *CA*(*i *+ 2), *CA*(*i *+ 3), *CA*(*i *+ 4)))

where the subscript *c *refers to correlation energies. If *φ*_*i *_is the dihedral defined by *CA*(*i*) - *CA*(*i *+ 1) - *CA*(*i *+ 2) - *CA*(*i *+ 3), the correlation energy for the two-dimensional bin (*m*, *n*) is defined as:

−log⁡(p(m,n)p(m)p(n))
 MathType@MTEF@5@5@+=feaafiart1ev1aaatCvAUfKttLearuWrP9MDH5MBPbIqV92AaeXatLxBI9gBaebbnrfifHhDYfgasaacH8akY=wiFfYdH8Gipec8Eeeu0xXdbba9frFj0=OqFfea0dXdd9vqai=hGuQ8kuc9pgc9s8qqaq=dirpe0xb9q8qiLsFr0=vr0=vr0dc8meaabaqaciaacaGaaeqabaqabeGadaaakeaacqGHsislcyGGSbaBcqGGVbWBcqGGNbWzcqGGOaakdaWcaaqaaiabdchaWjabcIcaOiabd2gaTjabcYcaSiabd6gaUjabcMcaPaqaaiabdchaWjabcIcaOiabd2gaTjabcMcaPiabdchaWjabcIcaOiabd6gaUjabcMcaPaaacqGGPaqkaaa@433A@

where *m *and *n *refer to angles *φ*_*i *_and *φ*_*i *+ 1_, respectively and the probability *p*(*m*, *n*) is computed over all pairs *φ*_*i*_, *φ*_*i *+ 1_.

The energetics of non-bonded interactions among the centers of interaction in the reduced model is described by the sum of three terms:

*E*_*nb *_= *ix*) *E*(*d*(*CM*(*i*) - *CM*(*j*)))_|*j*-*i*|>1 _+

*x*) *E*(*d*(*CA*(*i*) - *CA*(*j*)))_|*j*-*i*|>2 _+

*xi*) *E*(*d*(*CA*(*i*) - *CM*(*j*)))_|*j*-*i*|>1 _

The energy of the *k*^*th *^bin of the distribution is computed as:

E(k)=−log⁡(Nobs(k)Nexp(k))
 MathType@MTEF@5@5@+=feaafiart1ev1aaatCvAUfKttLearuWrP9MDH5MBPbIqV92AaeXatLxBI9gBaebbnrfifHhDYfgasaacH8akY=wiFfYdH8Gipec8Eeeu0xXdbba9frFj0=OqFfea0dXdd9vqai=hGuQ8kuc9pgc9s8qqaq=dirpe0xb9q8qiLsFr0=vr0=vr0dc8meaabaqaciaacaGaaeqabaqabeGadaaakeaacqWGfbqrcqGGOaakcqWGRbWAcqGGPaqkcqGH9aqpcqGHsislcyGGSbaBcqGGVbWBcqGGNbWzcqGGOaakdaWcaaqaaiabd6eaonaaBaaaleaacqWGVbWBcqWGIbGycqWGZbWCaeqaaOGaeiikaGIaem4AaSMaeiykaKcabaGaemOta40aaSbaaSqaaGqaciab=vgaLjab=Hha4jab=bhaWbqabaGccqGGOaakcqWGRbWAcqGGPaqkaaGaeiykaKcaaa@49D2@

where *N*_*exp*_(*i*) and *N*_*obs *_are the number of counts of centers of interaction expected and actually found in the *k*^*th *^bin of the distribution, respectively. The expected number of counts in a given bin (*N*_*exp*_) requires a proper treatment, because proteins are finite systems and different types of center of interactions have different dimensions. The finite size of proteins will result in a decay of the counts at long distances. In the absence of specific interactions and neglecting excluded volume or correlation effects, the expected number of counts within any given distance range *r *- Δr2
 MathType@MTEF@5@5@+=feaafiart1ev1aaatCvAUfKttLearuWrP9MDH5MBPbIqV92AaeXatLxBI9gBaebbnrfifHhDYfgasaacH8akY=wiFfYdH8Gipec8Eeeu0xXdbba9frFj0=OqFfea0dXdd9vqai=hGuQ8kuc9pgc9s8qqaq=dirpe0xb9q8qiLsFr0=vr0=vr0dc8meaabaqaciaacaGaaeqabaqabeGadaaakeaadaWcaaqaaiabfs5aejabdkhaYbqaaiabikdaYaaaaaa@3081@ to *r *+ Δr2
 MathType@MTEF@5@5@+=feaafiart1ev1aaatCvAUfKttLearuWrP9MDH5MBPbIqV92AaeXatLxBI9gBaebbnrfifHhDYfgasaacH8akY=wiFfYdH8Gipec8Eeeu0xXdbba9frFj0=OqFfea0dXdd9vqai=hGuQ8kuc9pgc9s8qqaq=dirpe0xb9q8qiLsFr0=vr0=vr0dc8meaabaqaciaacaGaaeqabaqabeGadaaakeaadaWcaaqaaiabfs5aejabdkhaYbqaaiabikdaYaaaaaa@3081@ depends on the density of the relevant centers of interaction in the dataset proteins and is proportional to the spherical shell volume around the reference center:

Nexp(r−Δr2,r+Δr2)=a∗r2∗Δr
 MathType@MTEF@5@5@+=feaafiart1ev1aaatCvAUfKttLearuWrP9MDH5MBPbIqV92AaeXatLxBI9gBaebbnrfifHhDYfgasaacH8akY=wiFfYdH8Gipec8Eeeu0xXdbba9frFj0=OqFfea0dXdd9vqai=hGuQ8kuc9pgc9s8qqaq=dirpe0xb9q8qiLsFr0=vr0=vr0dc8meaabaqaciaacaGaaeqabaqabeGadaaakeaacqWGobGtdaWgaaWcbaacbiGae8xzauMae8hEaGNae8hCaahabeaakiabcIcaOiabdkhaYjabgkHiTmaalaaabaGaeuiLdqKaemOCaihabaGaeGOmaidaaiabcYcaSiabdkhaYjabgUcaRmaalaaabaGaeuiLdqKaemOCaihabaGaeGOmaidaaiabcMcaPiabg2da9iabdggaHjabgEHiQiabdkhaYnaaCaaaleqabaGaeGOmaidaaOGaey4fIOIaeuiLdqKaemOCaihaaa@4AB8@

**Figure 4:**
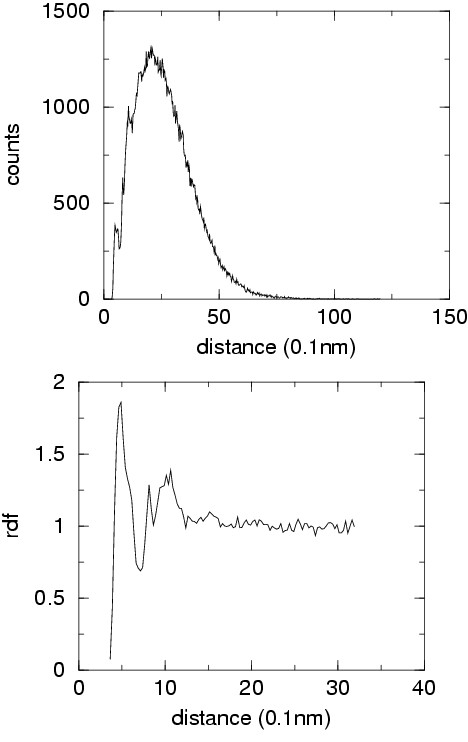
Original histogram of counts for the distances between the Ile and Ala sidechain centers of interaction (bin width = 0.25 Å) (upper panel). Computed radial distribution function (lower panel).

The finite size of proteins makes larger distances less and less probable. We found that a simple exponential damping factor describes this effect fairly well. For this reason the expected number of counts in a bin at a given distance *r *may be expressed as:

*N*_*exp*_(*r*) = a * *r*^2 ^* *exp*(-(*r*/*b*)^*c*^) * Δ*r*

where *a*, *b *and *c *are fitting parameters. These parameters take account of both the density of the interaction centers and finite size of proteins. Assuming that the interactions among amino acids are short ranged, as it is usally assumed, the fitting parameters *a*, *b *and *c *may be estimated from the observed distributions at large distances (say at more than 16 Å). This was done here and the effectiveness of this approach is shown in Figure 4 where the original counts' distribution and the computed radial distribution function are shown. This choice for the reference state corresponds to a finite size randomly collapsed protein. It has the advantage, over other possible definition of the reference state, of taking into account in a straightforward way the finite size of proteins and the different size of amino acids. Note that the definition of average amino acid interactions would be problematic in the presence of very different radial distributions.

### Computation of conformational energies

In order to properly evaluate conformational energies, some energy minimization should be performed, because the model at hand might have been not refined and minor deviations from standard geometry could result in large unfavorable energy terms. We assumed that any given conformation could relax toward lower energies, this is even more true when simplified models are taken into account. A dislocation of 10 degrees was therefore allowed as far as angles and pseudoangles are concerned, as well as a variation of 0.05 Å for the distance *d*(*CA*(*i*), *CA*(*i *+ 1)) and of 0.2 Å for the distance *d*(*CA*(*i*), *CM*(*i*)). We remark that no real movement of the structure is allowed, but rather we take the minimum energy value in the allowed range for every contribution to the energy. This virtual relaxation of the structure is able to reduce the dependence of the energy on the simplification procedure and on building details.

Another problem concerns the exact weigthing of the different contributions. Energy terms connected with the covalent structure of the reduced model have been treated ad hoc, in order to avoid that a structure built with all angles and lengths at their energy minimum values could get a very low energy. All these contributions (terms *i *to *vi *in equation 6) have been reassigned as follows:

E={0if E<E¯+σEE−E¯−σEσE(1.0−exp⁡E−E¯−σEσE)if E≥E¯+σE
 MathType@MTEF@5@5@+=feaafiart1ev1aaatCvAUfKttLearuWrP9MDH5MBPbIqV92AaeXatLxBI9gBaebbnrfifHhDYfgasaacH8akY=wiFfYdH8Gipec8Eeeu0xXdbba9frFj0=OqFfea0dXdd9vqai=hGuQ8kuc9pgc9s8qqaq=dirpe0xb9q8qiLsFr0=vr0=vr0dc8meaabaqaciaacaGaaeqabaqabeGadaaakeaacqWGfbqrcqGH9aqpdaGabaqaauaabeaaciaaaeaacqaIWaamaeaacqqGPbqAcqqGMbGzcqqGGaaicqWGfbqrcqGH8aapcuWGfbqrgaqeaiabgUcaRGGaciab=n8aZnaaBaaaleaacqWGfbqraeqaaaGcbaWaaSaaaeaacqWGfbqrcqGHsislcuWGfbqrgaqeaiabgkHiTiab=n8aZnaaBaaaleaacqWGfbqraeqaaaGcbaGae83Wdm3aaSbaaSqaaiabdweafbqabaaaaOGaeiikaGIaeGymaeJaeiOla4IaeGimaaJaeyOeI0IagiyzauMaeiiEaGNaeiiCaa3aaSaaaeaacqWGfbqrcqGHsislcuWGfbqrgaqeaiabgkHiTiab=n8aZnaaBaaaleaacqWGfbqraeqaaaGcbaGae83Wdm3aaSbaaSqaaiabdweafbqabaaaaOGaeiykaKcabaGaeeyAaKMaeeOzayMaeeiiaaIaemyrauKaeyyzImRafmyrauKbaebacqGHRaWkcqWFdpWCdaWgaaWcbaGaemyraueabeaaaaaakiaawUhaaaaa@64E7@

where E¯
 MathType@MTEF@5@5@+=feaafiart1ev1aaatCvAUfKttLearuWrP9MDH5MBPbIqV92AaeXatLxBI9gBaebbnrfifHhDYfgasaacH8akY=wiFfYdH8Gipec8Eeeu0xXdbba9frFj0=OqFfea0dXdd9vqai=hGuQ8kuc9pgc9s8qqaq=dirpe0xb9q8qiLsFr0=vr0=vr0dc8meaabaqaciaacaGaaeqabaqabeGadaaakeaacuWGfbqrgaqeaaaa@2DD7@ is the average energy contribution per residue and *σ*_*E *_is the standard deviation in the top500H dataset. Since there are eleven different terms contributing the energy we decided to group together the covalent terms, but considered separately the dihedral term, the correlation term and the three non-bonded terms, and apply different weights to this terms. Setting the weights of the covalent term to one we tested combinatorially weights 0.5, 1, 2, 4, 8 on all other terms. The set of all multiple decoy sets in the Decoys'R'us database were tested and the performance of the weighting scheme was judged by average RMSD from native of the lowest energy model and by the average Z-score of the native structure. The final chosen weights were of 1 for the covalent, the dihedral and the correlation terms, and 8, 4 and 1 for CM-CM, CA-CA and CM-CA non-bonded interactions, respectively. The decoy sets more sensitive to the choice of weights was the semfold decoy set containing the largest number of decoys.

### Performance assessment: decoy sets and quality measures

In order to test extensively the performance of the model and associated energy function we considered all the decoy sets in the multiple category in the Decoys'R'us database [58]. These decoys have peculiar features and are representative of different realistic simulation scenarios. The potential function has been also tested in the model quality assessment program category of prediction at CASP7 (see e. g. ref. [69]). Five performance measures are considered for evaluation of the performance of the model [72].

1. *rank native*, the ranking of the native structure among the decoys. Ideally this should be 1, but for simplified models it might be that native-like models score even better than native structure.

2. *RMSD*, the RMSD of the best scoring conformation. This is a direct assessment of the quality of the reduced model and the associated energy function, provided that decoys are well constructed and that there are native-like decoys in the set.

3. *cc*, the correlation coefficient between energy and RMSD. This may be low if the set is composed mostly of misfolded structures.

4. *Z-score*, the Z-score of the native structure in the decoys set. This parameter should measure the discriminative power of the potential. It strongly depends on the quality of the decoys in the set.

5. *F.E*., the Fraction Enrichment, that is the percentage of the top 10% lowest RMSD structures that are found also in the top 10% best scoring ones.

## Availability and requirements

Parameters for the potential presented here are available at the URL .

## Authors' contributions

FF conceived the project, wrote part of the code used, performed the tests and analyses reported in the paper. LP wrote part of the code used and performed tests and analyses. LB, AD and GG tested the scoring function using ab-initio protein prediction programs suggesting improvements to the energy functions and provided discussion on specific issues of the energy function. AC, GE and PV set up the calculations with Rosetta and provided discussion on specific issues of the energy function in view of potential applications. All authors read and approved the final manuscript.

## Figures and Tables

**Table 1:** Performance evaluation of the energy function on 4state_reduced decoy set

Decoy set	PDB id.	rank native	RMSD	Z-score	cc	F.E.
4state_reduced	1ctf	1/631	0.0	-3.4	0.59	58.7
4state_reduced	**1r69**	1/676	0.0	-4.0	0.62	47.8
4state_reduced	1sn3	1/660	0.0	-3.6	0.36	34.8
4state_reduced	**2cro**	1/674	0.0	-3.2	0.69	55.2
4state_reduced	3icb	1/654	0.0	-2.9	0.76	70.8
4state_reduced	4pti	1/687	0.0	-3.1	0.40	33.8
4state_reduced	4rxn	667/677	2.1	2.5	0.48	46.3

**Table 2:** Performance evaluation of the energy function on fisa decoy set

Decoy set	PDB id	rank native	RMSD	Z-score	cc	F.E.
fisa	**1fc2-C**	1/501	0.0	-6.6	0.11	12.0
fisa	**1hdd-C**	1/501	0.0	-8.4	0.24	16.0
fisa	**2cro**	1/501	0.0	-7.3	0.17	22.0
fisa	4icb	1/500	0.0	-9.3	0.23	22.0

**Table 3:** Performance evaluation of the energy function on fisa_casp3 decoy set

Decoy set	PDB id.	rank native	RMSD	Z-score	cc	F.E.
fisa_casp3	**1bg8-A**	1/1201	0.0	-4.5	0.26	28.3
fisa_casp3	**1bl0**	1/972	0.0	-3.1	-0.09	7.2
fisa_casp3	**1eh2**	1/2414	0.0	-3.0	0.13	18.3
fisa_casp3	**1jwe**	1/1408	0.0	-5.6	0.10	15.0
fisa_casp3	**l30**	1/1401	0.0	-5.6	0.06	13.6
fisa_casp3	**smd3**	1/1201	0.0	-4.4	0.06	14.2

**Table 4:** Performance evaluation of the energy function on hg_structal decoy set

Decoy set	PDB id.	rank native	RMSD	Z-score	cc	F.E.
hg_structal	**1ash**	1/30	0.0	-2.0	0.62	66.7
hg_structal	1bab-B	3/30	0.8	-1.4	0.83	66.7
hg_structal	**1col-A**	1/30	0.0	-4.8	0.73	33.3
hg_structal	1cpc-A	1/30	0.0	-3.4	0.61	33.3
hg_structal	1ecd	28/30	1.5	1.8	0.57	33.3
hg_structal	1emy	3/30	0.8	-1.9	0.81	100.0
hg_structal	1flp	3/30	1.9	-1.5	0.44	33.3
hg_structal	1gdm	1/30	0.0	-3.0	0.82	100.0
hg_structal	1hbg	1/30	0.0	-2.2	0.54	33.3
hg_structal	1hbh-A	2/30	1.1	-1.6	0.87	33.3
hg_structal	1hbh-B	5/30	1.0	-1.1	0.80	33.3
hg_structal	1hda-A	2/30	0.5	-1.5	0.92	66.7
hg_structal	1hda-B	1/30	0.0	-1.4	0.84	100.0
hg_structal	1hlb	9/30	2.9	-0.4	0.55	33.3
hg_structal	1hlm	30/30	4.0	2.3	0.21	33.3
hg_structal	1hsy	5/30	0.9	-1.3	0.82	66.7
hg_structal	1ith-A	1/30	0.0	-2.6	0.78	66.7
hg_structal	1lht	30/30	0.8	3.0	0.41	66.7
hg_structal	1mba	1/30	0.0	-1.7	0.72	33.3
hg_structal	1mbs	30/30	1.8	2.2	0.52	66.7
hg_structal	1myg-A	1/30	0.0	-2.0	0.82	66.7
hg_structal	1myj-A	2/30	0.6	-1.8	0.86	66.7
hg_structal	1myt	1/30	0.0	-2.3	0.72	66.7
hg_structal	2dhb-A	8/30	0.8	-0.8	0.85	66.7
hg_structal	2dhb-B	8/30	1.0	-0.7	0.83	66.7
hg_structal	2lhb	1/30	0.0	-2.6	0.71	33.3
hg_structal	2pgh-A	5/30	1.0	-1.2	0.91	33.3
hg_structal	2pgh-B	7/30	0.8	-0.8	0.85	33.3
hg_structal	4sdh-A	1/30	0.0	-3.6	0.70	33.3

**Table 5:** Performance evaluation of the energy function on ig_structal decoy set

Decoy set	PDB id.	rank native	RMSD	Z-score	cc	F.E.
ig_structal	1bbd	57/61	2.1	1.4	0.11	0.0
ig_structal	1bbj	61/61	1.2	3.6	0.28	0.0
ig_structal	1dbb	57/61	1.4	1.1	0.43	0.0
ig_structal	1dfb	47/61	2.0	0.4	0.48	0.0
ig_structal	1dvf	28/61	0.7	-0.3	0.43	16.7
ig_structal	1eap	33/61	2.1	-0.1	0.39	16.7
ig_structal	1fai	4/61	2.2	-1.3	0.62	33.3
ig_structal	1fbi	61/61	1.8	6.0	-0.01	16.7
ig_structal	1fgv	50/61	1.4	0.8	0.48	0.0
ig_structal	1fig	61/61	2.0	6.1	-0.03	16.7
ig_structal	1flr	6/61	1.9	-1.1	0.49	16.7
ig_structal	1for	59/61	2.1	2.2	0.36	0.0
ig_structal	1fpt	29/61	1.7	-0.2	0.51	0.0
ig_structal	1frg	5/61	2.1	-1.5	0.37	33.3
ig_structal	1fvc	54/61	4.1	0.9	0.07	16.7
ig_structal	1fvd	13/61	1.8	-0.7	0.42	0.0
ig_structal	1gaf	35/61	1.7	0.1	0.43	33.3
ig_structal	1ggi	58/61	1.7	1.2	0.39	16.7
ig_structal	1gig	5/61	1.7	-1.4	0.43	66.7
ig_structal	1hil	5/61	0.9	-1.1	0.53	33.3
ig_structal	1hkl	25/61	1.6	-0.3	0.49	16.7
ig_structal	1iai	51/61	1.2	1.1	0.44	33.3
ig_structal	1ibg	40/61	4.1	0.3	0.14	0.0
ig-structal	1igc	17/61	1.0	-0.6	0.49	16.7
ig_structal	1igf	45/61	1.8	0.4	0.50	16.7
ig_structal	1igi	23/61	3.3	-0.4	0.34	0.0
ig_structal	1igm	14/61	1.5	-0.8	0.52	33.3
ig_structal	1ikf	10/61	2.5	-0.9	0.50	16.7
ig_structal	1ind	2/61	1.2	-1.9	0.57	50.0
ig_structal	1jel	59/61	1.3	2.2	0.36	33.3
ig_structal	1jhl	46/61	2.9	0.5	0.32	33.3
ig_structal	1kem	16/61	2.0	-0.6	0.52	16.7
ig_structal	1mam	39/61	2.0	0.2	0.26	16.7
ig_structal	1mcp	27/61	2.1	-0.3	0.27	0.0
ig_structal	1mfa	60/61	3.4	1.4	-0.02	0.0
ig_structal	1mlb	55/61	1.3	0.9	0.36	16.7
ig_structal	1mrd	61/61	2.9	5.3	-0.21	0.0
ig_structal	1nbv	34/61	2.0	0.1	0.39	33.3
ig_structal	1ncb	55/61	1.3	1.0	0.45	33.3
ig_structal	1ngq	59/61	1.5	1.8	0.27	16.7
ig_structal	1nmb	34/61	4.4	-0.1	0.15	0.0
ig_structal	1nsn	61/61	2.0	3.2	0.20	16.7
ig_structal	1opg	49/61	1.7	0. 6	0.46	33.3
ig_structal	1plg	32/61	1.5	-0.2	0.57	0.0
ig_structal	1rmf	57/61	1.7	1.7	0.35	16.7
ig_structal	1tet	51/61	1.5	0.7	0.44	0.0
ig_structal	1ucb	18/61	1.6	-0.6	0.54	33.3
ig_structal	1vfa	2/61	2.7	-1.6	0.33	16.7
ig_structal	1vge	31/61	3.9	-0.0	0.10	16.7
ig_structal	1yuh	61/61	1.9	2.4	0.06	33.3
ig_structal	2cgr	49/60	1.4	0.5	0.45	16.7
ig_structal	2fb4	4/61	1.7	-1.5	0.43	50.0
ig_structal	2fbj	20/61	1.4	-0.4	0.48	0.0
ig_structal	2gfb	4/61	2.0	-1.5	0.40	50.0
ig_structal	3hfl	59/61	4.2	2.7	-0.29	0.0
ig_structal	3hfm	59/61	1.6	1.9	0.38	0.0
ig_structal	6fab	29/61	1.4	-0.1	0.48	0.0
ig_structal	7fab	57/61	2.0	1.2	0.36	33.3
ig_structal	8fab	44/61	5.3	0.4	-0.01	0.0

**Table 6:** Performance evaluation of the energy function on ig_structal_hires decoy set

Decoy set	PDB id.	rank native	RMSD	Z-score	cc	F.E.
ig_structual_hires	1dvf	11/20	0.7	-0.2	0.50	50
ig_structual_hires	1fgv	18/20	1.4	1.0	0.39	0
ig_structual_hires	1flr	2/20	2.0	-0.9	0.71	50
ig_structual_hires	1fvc	19/20	1.7	1.1	-0.13	0
ig_structual_hires	1gaf	13/20	1.6	0.2	0.46	0
ig_structual_hires	1hil	2/20	2.5	-0.7	0.54	50
ig_structual_hires	1ind	1/20	0.0	-1.5	0.49	50
ig_structual_hires	1kem	9/20	2.0	-0.3	0.59	0
ig_structual_hires	1mfa	19/20	3.2	0.7	-0.04	0
ig_structual_hires	1mlb	18/20	3.7	0.9	0.35	0
ig_structual_hires	1nbv	14/20	2.2	0.2	0.41	0
ig_structual_hires	1opg	19/20	1.9	1.0	0.20	0
ig_structual_hires	1vfa	2/20	2.7	-1.2	0.33	50
ig_structual_hires	1vge	13/20	4.1	0.0	-0.024	0
ig_structual_hires	2cgr	15/20	1.4	0.3	0.47	0
ig_structual_hires	2fb4	3/20	1.7	-1.0	0.48	50
ig_structual_hires	2fbj	10/20	1.4	-0.2	0.56	0
ig_structual_hires	6fab	12/20	2.1	0.0	0.46	0
ig_structual_hires	7fab	19/20	1.8	0.9	0.34	50
ig_structual_hires	8fab	13/20	5.6	0.4	-0.13	0

**Table 7:** Performance evaluation of the energy function on lattice_ssfit set

Decoy set	PDB id.	rank native	RMSD	Z-score	cc	F.E.
lattice_ssfit	**1beo**	1/1998	0.0	-5.6	0.08	12.6
lattice_ssfit	1ctf	1/2001	0.0	-6.0	0.03	16.0
lattice_ssfit	**1dkt-A**	1/1999	0.0	-3.1	-0.01	11.6
lattice_ssfit	1fca	1/2001	0.0	-4.7	0.04	9.0
lattice_ssfit	**1nkl**	1/1998	0.0	-4.1	0.01	14.1
lattice_ssfit	1pgb	1/2000	0.0	-4.7	0.04	10.5
lattice_ssfit	**1trl-A**	1/2000	0.0	-3.6	0.02	10.0
lattice_ssfit	4icb	1/2000	0.0	-4.4	-0.00	15.5

**Table 8:** Performance evaluation of the energy function on lmds decoy set

Decoy set	PDB id.	rank native	RMSD	Z-score	cc	F.E.
lmds	**1b0n-B**	1/498	0.0	-3.3	0.05	18.4
lmds	**1bba**	501/501	4.5	21.4	-0.23	10.0
lmds	1ctf	1/498	0.0	-3.4	0.31	2.0
lmds	1dtk	2/216	6.3	-2.5	0.21	33.3
lmds	**1fc2-C**	53/501	5.4	-1.3	0.17	24.0
lmds	1igd	1/501	0.0	-4.0	0.10	6.0
lmds	1shf-A	1/438	0.0	-5.3	0.11	11.6
lmds	**2cro**	1/501	0.0	-7.7	0.13	20.0
lmds	2ovo	1/348	0.0	-3.2	0.11	5.9
lmds	4pti	1/344	0.0	-3.5	0.02	14.7

**Table 9:** Performance evaluation of the energy function on semfold decoy set

Decoy set	PDB id.	rank native	RMSD	Z-score	cc	F.E.
semfold	1ctf	1/11402	0.0	-4.7	0.13	19.7
semfold	**1e68**	5/11361	4.5	-2.3	0.09	21.0
semfold	**1eh2**	69/11442	0.3	-2.3	0.06	13.3
semfold	**1khm**	10/21081	11.4	-3.0	0.02	11.3
semfold	**1nkl**	4/11662	0.2	-3.5	0.09	19.4
semfold	1pgb	1/11282	0.0	-3.1	0.03	12.7

**Table 10:** Performance evaluation of the energy function on vhp_mcmd decoy set

Decoy set	PDB id.	rank native	RMSD	Z-score	cc	F.E.
vhp_mcmd	**1vii**	2364/6256	2.6	-0.6	0.57	35.0

**Table 11:** Comparison of different model quality assessment programs

	4state_reduced		lattice_ssfit		Imds	
MQAP	Rank native	Z-score	Rank native	Z-score	Rank native	Z-score

PC2CA	6/7	-2.5	8/8	-4.5	7/10	-1.3
ProQ	5/7	-4.1	7/8	-12.1	4/10	-3.7
Prosa II	5/7	-2.7	8/8	-5.6	6/10	-2.5
Verify3D	4/7	-2.6	7/8	-4.5	2/10	-1.4
AKBP	7/7	-3.2	8/8	-6.6	3/10	-0.5
DFIRE	6/7	-3.5	8/8	-9.5	7/10	-0.9
RAPDF	7/7	-3.0	8/8	-7.2	3/10	+0.5
FRST	7/7	-4.4	8/8	-6.7	6/10	-3.5
